# Discovery of a Novel Inhibitor Structure of *Mycobacterium tuberculosis* Isocitrate Lyase

**DOI:** 10.3390/molecules27082447

**Published:** 2022-04-11

**Authors:** Changyuan Duan, Qihua Jiang, Xue Jiang, Hongwei Zeng, Qiaomin Wu, Yang Yu, Xiaolan Yang

**Affiliations:** 1 Key Laboratory of Medical Laboratory Diagnostics of the Education Ministry, College of Laboratory Medicine, Chongqing Medical University, No. 1, Yixueyuan Road, Yuzhong Dist, Chongqing 400016, China; yyduan0428@163.com (C.D.); jjiangdeyouxiang@163.com (X.J.); macezhuan03922@163.com (H.Z.); wuqiaomin4869@163.com (Q.W.); 15008445928@163.com (Y.Y.); 2 College of Pharmacy, Chongqing Medical University, Chongqing 400016, China; 100877@cqmu.edu.cn

**Keywords:** tuberculosis (TB), virtual screening, isocitrate lyase, molecular docking

## Abstract

Tuberculosis remains a global threat to public health, and dormant *Mycobacterium tuberculosis* leads to long-term medication that is harmful to the human body. *M. tuberculosis* isocitrate lyase (*Mt*ICL), which is absent in host cells, is a key rate-limiting enzyme of the glyoxylic acid cycle and is essential for the survival of dormant *M. tuberculosis*. The aim of this study was to evaluate natural compounds as potential *Mt*ICL inhibitors through docking and experimental verification. Screening of the TCMSP database library was done using Discovery Studio 2019 for molecular docking and interaction analysis, with the putative inhibitors of *Mt*ICL, 3-BP, and IA as reference ligands. Daphnetin (MOL005118), with a docking score of 94.8 and -CDOCKER interaction energy of 56 kcal/mol, was selected and verified on *Mt*ICL in vitro and *M. smegmatis*; daphnetin gave an IC_50_ of 4.34 μg/mL for the *Mt*ICL enzyme and an MIC value of 128 μg/mL against *M. smegmatis*, showing enhanced potential in comparison with 3-BP and IA. The interactions and essential amino acid residues of the protein were analyzed. In summary, natural daphnetin may be a promising new skeleton for the design of inhibitors of *Mt*ICL to combat dormant *M. tuberculosis*.

## 1. Introduction

Tuberculosis (TB) is caused by the lethal pathogen *Mycobacterium tuberculosis* (*Mtb*) and remains one of the deadliest infectious diseases worldwide [1]. Although the incidence of TB has declined, the increasing number of drug-resistant strains, especially multidrug-resistant (MDR) and super-drug-resistant (XDR) strains, have posed great challenges to the treatment of tuberculosis [2]. According to the WHO, 8.9–11 million people were infected with TB globally, with 1.1–1.2 million deaths occurring in 2019 [3]. Worldwide, there are approximately 500,000 cases of rifampicin-resistant TB (RR-TB), of which ~78% are MDR-TB; MDR/RR-TB occurs in 3.3% of new TB cases and 17.7% of treated TB cases. However, *Mtb* can live in the host for years without clinical manifestations, which is known as latent TB. TB has a latency period that is longer than that of any other infectious disease [4]. It is estimated that at least a quarter of the world’s population is latently infected by bacteria [4], of which approximately 5–15% will develop active TB when they become immunocompromised. The latent infection comprises approximately 90% of TB cases [5]. Treatment of latent TB infection is essential for controlling and eliminating TB worldwide to achieve the goal of the End TB Strategy by 2035. To date, the standard first-line treatment for drug-sensitive TB lasts for 6 months, including 2 months of rifampicin, isoniazid, ethambutol, and pyrazinamide, followed by rifampicin and isoniazid for approximately 4 months, for a complete eradication of active *Mtb* and to prevent dormant bacilli from developing into active TB. However, no drugs in the clinic are effective against dormant *Mtb*, except pyrazinamide, which is not used because of its high hepatotoxicity [6]. However, the treatment of drug-resistant TB requires more than 20 months [7]. Therefore, more effective anti-tuberculosis drugs are needed to fight latent TB and MDR/MDR-TB.

There are various mechanisms underlying the dormancy and survival of *Mtb*. In the host, phagocytized *Mtb* and those maintained in granuloma suffer from hypoxia, nutrient deprivation, limited carbon sources, and high concentrations of nitric oxide. Dormant *Mtb* can reinforce anaerobic metabolic pathways to accommodate hypoxia [8,9,10]. The glycolate cycle is an alternative pathway to the tricarboxylic acid cycle under hypoxia, which allows bacteria to grow by using glyoxalates or fatty acids as carbon sources and plays an important role in the survival of *Mtb* [4]. Isocitrate lyase (ICL) is a key rate-limiting enzyme involved in the glyoxylate cycle, bypassing several β-oxidation steps in the Krebs cycle to reversibly decompose isocitrate into succinic acid and glyoxylate and producing energy [11,12]. Additionally, the upregulated alanine dehydrogenase (Ald) catalyzes the conversion of pyruvate to alanine, glycine dehydrogenase catalyzes the conversion of glyoxyate to glycine, and NADH is oxidized to NAD [13]. Glyoxylate and pyruvate are provided by the glyoxylate cycle and the methylcitrate cycle, respectively, and are catalyzed by two isomers of isocitrate lyase (ICL1 and ICL2), which are encoded by the genes icl and aceA, respectively [14]. Deletion of either or both the ICL and aceA genes impairs *Mtb* survival in activated macrophages and reduces their virulence [15,16,17]. In the absence of ICL, *Mtb* did not survive in the latent TB model. Moreover, these two pathways are absent in the host. Therefore, *M. tuberculosis* isocitrate lyase (*Mt*ICL) is a potential drug target for latent bacteria that has attracted increasing research interest [4,6,18,19].

Most of the classical inhibitors of ICL are structural analogs of the products of ICL-catalyzed reactions, such as 3-bromopyruvic acid (3-BP) [20], an analog of glyoxylate, and itaconic acid (IA) [21] and 3-nitropropanoic acid (3-NP) [22], analogs of succinic acid. However, these inhibitors have weak inhibition ability and strong biological toxicity [14] and have not been applied clinically. Several potential ICL inhibitors based on high-throughput screening (HTS) have been reported, such as XHD-1/XHD-2 [23] and IMbI-3 [24] from the Chinese medicine compound library, synthetic compounds of 3-nitropropionamides with inhibition at nanomolar levels [25], heptapeptide [26] from the chemical synthesis library, and a phage peptide library.

Virtual screening (VS) provides a new approach and alternative to high-throughput screening, which can significantly save experimental costs. By target-based VS of small molecules against characteristic binding sites, the molecules were selected as hits with high binding ability to target according to docking scores and interaction analysis [27], and further, according to the favorable binding modes after molecular dynamic simulation. Finally, hits were verified by subsequent bioactivity tests. Recently, there have been reports of VS based on the *Mt*ICL structure for inhibitors with no enzymatic assays [28,29]. 

In this study, to mine a new skeleton for *Mt*ICL inhibitors from Chinese medicines, the TCMSP database (https://old.tcmsp-e.com/tcmsp.php, accessed on 13 November 2020), with a total of 12,811 natural compounds, was used for VS. First, based on the natural crystal structure of ICL (PDB:1F8M), the active site was determined for VS. Candidate compounds with docking scores over 80 and -CDOCKER interaction energies >50 kcal/mol were selected; their IC_50_ for *Mt*ICL and MIC against *M. smegmatis* were evaluated. Finally, MOL005118 (named as daphnetin hereafter), with potent inhibition and favorable pharmacokinetics, was found to be a promising new skeleton for *Mt*ICL inhibitors.

## 2. Results

### 2.1. Virtual Screening

In this study, 12,811 compounds were screened. The workflow is shown in Figure S1. First, 8784 compounds were selected from the preliminary screening following the Lipinski and Veber rules. Subsequently, rigid docking and flexible docking were performed in sequence on the compound library using two functional modules of LibDock and CDOCKER. LibDock was first applied for the fast loading of these compounds in rigid conformations to the binding pocket of *Mt*ICL according to the hotspot principle of target-ligand interaction. LibDock is suitable for the fast and accurate virtual screening of large-scale databases. X, Y, and Z were set as 18.621885, 39.857179, and 52.768100, respectively. The known *Mt*ICL inhibitors 3-BP and IA were used as reference ligands, and their scores after LibDock were 55.7 and 72.9, respectively. Small molecules with LibDock scores ≥80 were selected for flexible docking on CDOCKER with a CHARMm-based molecular dynamics (MD) algorithm [30]. CHARMm is the most widely used molecular dynamics field in computer simulations. It can perform energy calculations for either small or large molecules (proteins, nucleic acids, and sugars) including interaction and conformational energies, minimization, and dynamic behavior [31,32,33,34].

**Figure 1:**
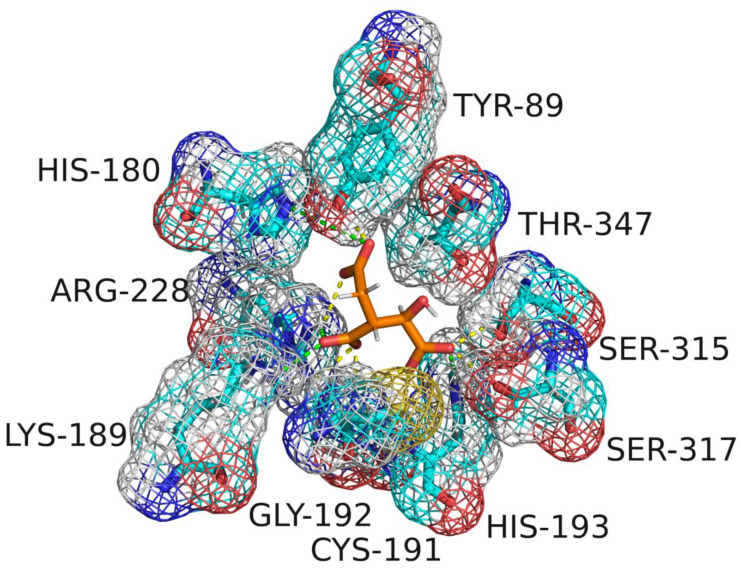
Active site of *Mt*ICL binding with isocitrate.

**Figure 2:**
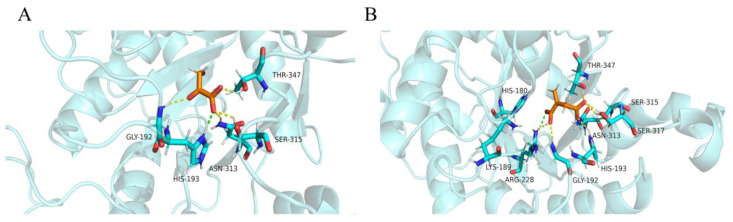
Diagram of interactions between *Mt*ICL and reference ligands. (**A**): 3-BP; (**B**): IA.

After CDOCKER docking, 60 compounds with a -CDOCKER interaction energy >50 kcal/mol were selected for the next step of manual selection. The mode of interaction of isocitrate as the natural substrate for *Mt*ICL was compared with the binding modes of the candidate compounds for *Mt*ICL. As shown in Figure 1, the oxygen atoms of the carbonyl and hydroxyl groups of isocitrate are the main hydrogen bond receptors, forming conventional hydrogen bonds with amino acid residues in the active sites, such as TYR89, GLY192, SER315, SER317, and THR347. In addition, HIS180, LYS189, HIS193, and ARG228 form electrostatic interactions with isocitrate. To simplify the picture, some amino acid residues with weak interaction forces are hidden. This is consistent with the reported situation of essential amino acid residues in the *Mt*ICL active site [35]. Similarly, consistent results are observed for the interactions of 3-BP and IA. As shown in Figure 2, GLY192, SER315, THR347, ASN313, and HIS193 of *Mt*ICL interact with the oxygen atoms of 3-BP. GLY192, HIS198, ASN313, SER315, SER317, and THR347 are key residues involved in hydrogen bonding. IA forms hydrogen bonds with GLY192, HIS193, ASN313, SER315, THR317, and THR347 of *Mt*ICL and forms electrostatic interactions with LYS189, HIS180, and ARG228. Depending on the -CDOCKER interaction energy >50 kcal/mol and the binding models consistent with those of the reference compounds to the essential amino acid residues of *Mt*ICL, as shown in the 2D diagrams (Table S1), 11 compounds with structures different from those natural substrate analogs were selected for further experimental verification (Table 1).

**Figure 3:**
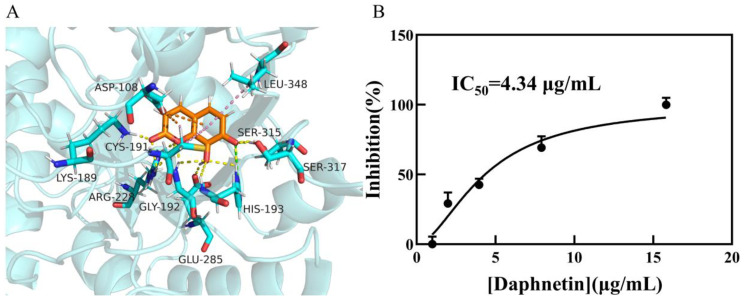
Interaction between daphnetin and *Mt*ICL. (**A**): Docking result of daphnetin with *Mt*ICL. Compounds: orange = daphnetin and blue = the amino acids interacting with daphnetin. Interacting bonds: yellow = hydrogen bond; green = electrostatic interaction; orange = π-anion; and pink = π-alkyl. (**B**): IC_50_ of daphnetin against *Mt*ICL.

**Figure 4:**
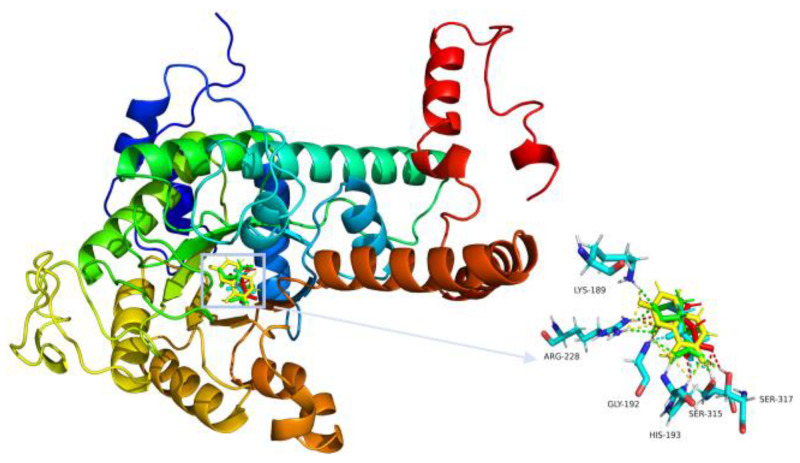
Overlapping diagram of four compounds within *Mt*ICL active sites. Red = IA; green = isocitric acid; yellow = daphnetin; and blue = 3-BP.

Daphnetin forms multiple interactions with *Mt*ICL residues, including π-alkyl interactions between the lactone ring and CYS191 and ASP108, and those between the phenol ring and CYS191, ASP108, and LEU348, in addition to the electrostatic attraction of HIS193 to the two oxygen atoms on the phenol moiety. In addition, a total of six amino acid residues (LYS189, GLY192, ARG228, GLU285, SER315, and SER317) form hydrogen bonds with carbonyl and hydroxide groups (Figure 3A). Other compounds also form hydrogen bonds with GLY192, ASN313, SER315, and other additional key residues (Table S1). The overlapping diagram shows a good structural similarity among the reference ligand, isocitrate, and daphnetin (Figure 4). Based on their interaction with *Mt*ICL, a certain number of hydrogen bond receptors, such as hydroxyl groups, are necessary for high inhibition potency against *Mt*ICL. These results can direct subsequent inhibitor development from daphnetin or others.

### 2.2. Enzyme Inhibitory Activity Assay 

The inhibitory potency against *Mt*ICL of the 11 hits and reference ligands was determined using UV spectrophotometry. In preliminary assays of 11 compounds at 333 μM or 500 μg/mL, 2 (Table 1) exhibited over 50% inhibition against *Mt*ICL, while the others had no significant inhibitory effect. The most potent daphnetin was further subjected to the dose-dependent inhibition assay, which gave an IC_50_ = 4.34 μg/mL (Figure 3B), while 3-BP and IA showed an IC_50_ of 63.6 ± 2.8 μM and 38.6 ± 0.8 μM, respectively. Daphnetin is a potent inhibitor of *Mt*ICL and a promising lead compound that is worth subsequent structural optimization.

### 2.3. Antibacterial Activity Assay

Streptomycin and levofloxacin as the positive controls showed MICs of 0.5 μg/mL and <0.125 μg/mL, respectively. The MICs of 3-BP and IA were higher than those of positive controls. The MIC of daphnetin was smaller than those of 3-BP and IA but still inferior to those of the positive controls (Table 2), which may be due to the limited permeability across the cell wall of *Mtb*. The other compounds showed no obvious antibacterial activity at 256 μg/mL.

### 2.4. Pharmacokinetics of Daphnetin 

ADMET calculations and toxicity predictions were performed for daphnetin and the two reference ligands, and the results are shown in Table 3 and Table 4. Daphnetin has good intestinal absorption and water solubility but low blood–brain barrier permeability. It has no inhibitory effect on CYP2D6, low non-binding with plasma proteins, and some hepatotoxic effects. In addition, rat oral LD_50_ and developmental toxicity potential (DTP) of daphnetin show low toxicity. The results of Ames show that none of the three compounds exhibit mutagenicity. As a result, daphnetin, with potent inhibition of *Mt*ICL but moderate antibacterial activity and hepatotoxicity, needs subsequent optimization or preparation as a prodrug to modify permeability and lessen potential toxicity.

### 2.5. Molecular Dynamics Simulation

**Figure 5:**
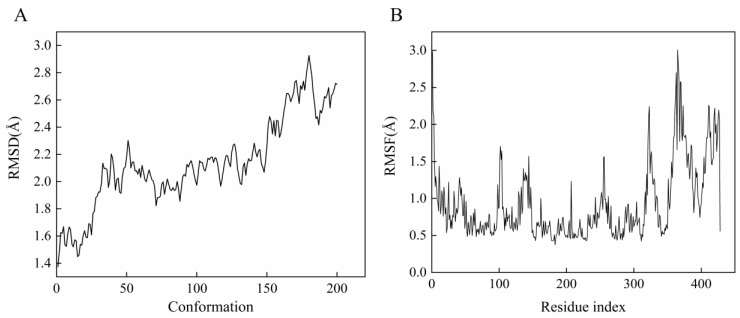
Results of MD simulations. (**A**): RMSF; (**B**): RMSD.

MD simulations were performed to determine the optimal binding conformation of daphnetin against *Mt*ICL. The NPT ensemble was set at 200 ps. After the equilibrium stage was achieved, the average temperature reached 300 K. A total of 200 conformations were generated, and the average RMSD value was 2.1375 (Figure 5A), supporting that the simulated system of the receptor complex was well balanced and had no obvious deviation from the initial structure. The RMSF values fluctuate significantly in the region comprising residues 310–398 (Figure 5B), suggesting that these regions play an important role in protein function.

## 3. Discussion

An efficient *Mt*ICL inhibitor, daphnetin was discovered from the small molecule database of Traditional Chinese Medicine (TCMSP) by virtual screening with the known receptor-binding site. Daphnetin is a coumarin derivative, 7,8-dithydroxycoumarin. Comparing with the reference ligands, daphnetin had a higher inhibitory capacity against *Mt*ICL (IC_50_ = 4.34 μg/mL) but a moderate antibacterial effect. This undesirable antibacterial effect might be caused by limited permeability across the cell wall of *Mtb*.

Daphnetin was the first new drug in China and is mainly derived from *Daphne Korean Nakai*. It has a variety of pharmacological effects, including cardiotonic, anti-inflammatory, antithrombotic, and antitumor effects [44,45,46,47]. Clinically, it is used to treat cardiovascular diseases such as coronary heart disease [48] and thromboangiitis obliterans [49]. It was mechanically reported that daphnetin has inhibitory activity on a variety of protein kinases, such as EGFR, PKA, and PKC, with IC_50_ values of 7.67 μM, 9.33 μM, and 25.01 μM, respectively [43], and also inhibition of the survival of hepatocellular carcinoma cells through regulating the Wnt/β-catenin signaling pathway [47]. Extensive researches related a lot of coumarin derivatives to a wide range of biological activities [50,51,52,53], suggesting that the specificity of coumarin derivatives is still an open question. However, no association of *Daphne Korean Nakai* or daphnetin with anti-TB activity has been reported. What is more, 4-methylumbelliferone and daphnetin are both coumarin derivatives, with the difference of a 4-methyl group on 4-methylumbelliferone and an 8-hydroxyl on daphnetin. However, in this study, as shown in Table 1, there was a large difference in the affinity against *Mt*ICL of daphnetin and 4-methylumbelliferone, suggesting that the 8-hydroxyl group of daphnetin may play a key role in the specific inhibition against *Mt*ICL. As a result, daphnetin, rather than 4-methylumbelliferone, is the potent inhibitor of *Mt*ICL.

*Mt*ICL is the key rate-limiting enzyme in the glyoxylate cycle, which is essential for the survival of dormant *Mtb* but is absent in the host; potent inhibitors of *Mt*ICL are still rare [25]. In this study, through the high-throughput virtual screening of a natural medicine library, daphnetin was found to have a strong inhibitory effect on *Mt*ICL and moderate anti-TB activity. According to pharmacokinetics results, daphnetin has good solubility in water, good intestinal absorption, no mutagenicity, and low toxicity. Moreover, the favorable interactions with *Mt*ICL, and thus high affinity against *Mt*ICL, support daphnetin as a promising lead compound worthy of structural modification to improve membrane permeability. The simple structure and low molecular weight of daphnetin facilitate the structural modification to improve membrane permeability and pharmacological activity. The insertion of a long alkyl moiety at the C3 position generally improved the lipophilicity beneficial for cell penetration [44], and also the substitution with triazole [54] and theophylline moieties [55] may improve its anti-TB activity. 

In conclusion, this study proposed daphnetin as a new lead skeleton targeting *Mt*ICL, which has the potential to be developed as an anti-tuberculosis drug targeting *Mt*ICL and effective against dormant *Mtb*. Further structural modifications are in progress.

## 4. Materials and Methods

### 4.1. Reagents

*Mt*ICL was expressed and purified as described previously [56]. The 3-BP was purchased from MCE (MedChemExpress, Shanghai, China). IA was purchased from Adamas (Adamas-beta, Shanghai, China). Daphnetin was purchased from Sigma-Aldrich (Sigma-Aldrich, St. Louis, MO, USA). Streptomycin was purchased from TCI (TCI Shangha Chemical industry development Co., Ltd., Shanghai, China). Levofloxacin came from Solarbio (Beijing Solarbio Science & Technology Co., Ltd., Beijing, China). Mycobacterium smegmatis mc2155 came from Guangdong Microbial Species Preservation Center. 

### 4.2. Methods

#### 4.2.1. Protein Preparation

The three-dimension crystal structure of *Mt*ICL bound with the inhibitor 3-BP (PDB:1F8M) was download from RCSB Protein Data Bank (http://www.rcsb.org, accessed on 5 January 2021). Protein structure was prepared by assigning partial charges, removing all the water molecules, and adding polar hydrogen atoms using Discovery Studio 2019 (DS) (BIOVIA Corp, San Diego, CA, USA). Considering the optimal pH of *Mt*ICL, the pH was set to 7.0. The sphere cavity accommodating 3-BP was defined as the active site for VS.

#### 4.2.2. Ligand Preparation 

A dataset of the Chinese medicine small molecules (12,811 compounds) was derived from TCMSP (https://old.tcmsp-e.com/tcmsp.php, accessed on 13 November 2020) in mol2 format and imported into DS. The library was screened by the Lipinski and Veber rules firstly. After that, the library was optimized by removing duplicates and enumerating isomers and tautomers, and their 3D conformations were generated. The pH values ranged from 6.5 to 8.5. Other parameters were set to default values.

#### 4.2.3. Virtual Screening 

From primary screening with LibDock module, small molecules with scores greater than or equal to 80 were selected. Then CDOCKER was used for precise docking. According to the resultant -CDOCKER interaction energy between each protein and compound conformation, compounds were selected with interaction energies >50 kcal/mol and structures other than those resembling the natural substrate citrate. Pymol 3.7 was used to visualize protein–ligand complexes. The same procedure was performed for the reference ligands.

#### 4.2.4. Enzyme Inhibitory Activity Assay

Enzyme inhibitory activity was evaluated by monitoring the decreasing rate of absorbance (V = ∆A/min) of enzyme reaction coupled with NADH at 340 nm [57]. *Mt*ICL at 0.5 μg and compounds at different final concentrations were added to a 300 μL enzymatic reaction system (5 mM MgCl_2_, 5 mM L-cysteine, 1 mM EDTA, pH 6.8). The mixture was incubated at 37 °C for 10 min, then 2 mM isocitrate was added and incubated for 20 min. Finally, 2U LDH and 0.2 mM NADH were added, and then the absorbance reduction at 340 nm in 3 min after 3 s mixing was monitored at intervals of 10 s (∆A) under room temperature. The enzyme activity was calculated according to the extinction coefficient of NADH (**ε** = 6.22 × 10^3^ mol^−^^1^·cm^−^^1^) [11]. A unit of the enzyme activity is defined as the amount of enzyme converting 1 μmol of substrate within 1 min. The percentage of inhibition of enzyme activity was calculated according to the following Equation (1):

(1) $$\mathrm{Inhibition}\;\mathrm{rate}\%=\frac{|V-V_{0}|}{V_{0}}\times 100$$

*V* indicates the reaction rate with the inhibitor, *V*_0_ indicates the reaction rate without the inhibitor. Graphpad prism 9.0 was used for non-linear fitting of “dose–response curves” for inhibitors to obtain an IC_50_ value of the compound against *Mt*ICL.

#### 4.2.5. Antibacterial Activity Assay

*M*. *smegmatis* Mc2155 is highly homologous to *Mtb*, but it grows rapidly and is noninfectious. Therefore, the compounds obtained by virtual screening were tested against *M. smegmatis* C2155 by mini-broth dilution test. *M. smegmatis* was cultured in Middlebrook 7H9 with OADC (10% Tween-80, 20% glycerol) and grew at 37 °C to a McTurbidities of about 0.5. *M. smegmatis* in culture was diluted at 1:1000 (about 1 × 10^5^ CFU/mL). The initial solution of each compound in dimethyl sulfoxide (DMSO) or sterile deionized water was in a 2-fold continuous dilution in a 96-well microplate containing 0.1 mL 7H9 medium. After that, 0.1 mL diluted bacterial solution was added and cultured at 37 °C for at least 48 h, and then the colony growth was observed. Positive control without drugs and negative control without bacteria were set up. Visual minimum inhibitory concentration (Visual MIC) was defined as the minimum concentration of drug without bacterial growth on the microplate.

#### 4.2.6. Pharmacokinetic Prediction and Dynamics Simulation of Hits 

To evaluate the druggability properties of candidate hits, ADMET calculations and toxicity predictions were performed using the Calculate Molecular Properties module in DS. ADMET can contribute to the early elimination of compounds with poor performance in ADMET and avoid the later waste of the huge financial resources and manpower required for structural modification. The ADMET can be calculated as follows: aqueous solubility, blood–brain barrier penetration, CYP2D6 binding, hepatotoxicity, intestinal absorption, and plasma protein binding. Toxicity prediction aspects included Ames, Rat oral LD50 and others. Additionally, 3-BP and IA were set as controls.

Molecular dynamics (MD) simulation in NPT thermodynamic ensemble was performed based on the CHARMm36 force field. The solvent environment was added as normal saline; minimization of 1000 steps was performed using the steepest descent method, and the thermal equilibrium temperature was set at 300 K. 

### 4.3. Statistical Analysis

All experiments were repeated three times. The results were expressed as mean ± standard deviation ($\bar{x}\pm s$).

## Figures and Tables

**Table 1:** Characterization of the selected compounds.

Compound	Structure	Inhibition Rate %	LibDock Score	-Interaction Energy (kcal/mol)	Natural Resource	Reported Target Name
L-Ascorbic acid	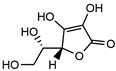	20.2 ^a^	97.1	60.0	*Ginkgo Semen, Herba Patriniae, Corayceps, etc.*	Cav3.2 channels [36]
4-Methylumbelliferone	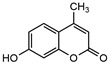	25.6 ^a^	84.5	55.4	*Olibanun*	Hyaluronic acid [37]
Quinic acid	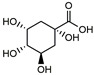	46.0 ^a^	99.8	52.7	*Boehmeriae Rhizoma Et Radix*	-
L-Shikimic acid	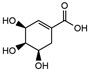	17.4 ^a^	94.4	52.3	*Anisi Stellati Fructus*	-
Chelidonic acid	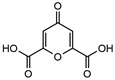	18.5 ^a^	95.3	52.2	*Chelidonium majus L.*	NF-κB [38]
Gallic acid	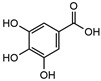	28.8 ^a^	98.6	51.6	*Palm leaf rhubarb, eucalyptus urophylla, dogwood, etc.*	COX-2 [39]
Phosphatidic acid	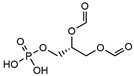	34.1 ^b^	99.8	57	*Angelicae Sinensis Radix and Panacis Quinquefolii Radix*	-
Methyl gallate	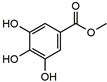	27.4 ^b^	95.7	54.1	*Paeoniae Radix Alba, Radix Paeoniae Rubra, Canavaliae Semen, etc.*	Bacterial [40]
3-*O*-Ethy-L-ascorbic acid	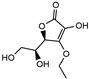	21.9 ^b^	93.9	52.9	*Hippophae Fructus and Sapindi Mukorossiperic Arpium*	Tyrosinase [41]
4-Deoxypyridoxine 5′-phosphate	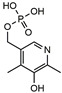	3.3 ^b^	93.2	55.6	*Hippophae Fructus*	Sphingosine 1-phosphate [42]
Daphnetin	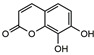	100 ^b^	94.8	56	*Daphne Korean Nakai*	EGFR, PKA, and PKC [43]
3-BP	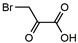	63.6 ± 2.8 (μM) ^c^	55.7	41	-	*Mt*ICL [20]
IA	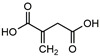	38.6 ± 0.8 (μM) ^c^	72.9	54	-	*Mt*ICL [21]

^a^ The final concentration of compounds was 333 μM. ^b^ The final concentration of compounds was 500 μg/mL. ^c^ IC_50_.

**Table 2:** Antibacterial activity of three compounds and control drugs.

Compound	MIC (μg/mL)
Daphnetin	128
3-BP	256
IA	>256
Streptomycin	0.5
Levofloxacin	<0.125

**Table 3:** The results of ADMET calculations.

Compound	Absorption Level	Solubility Level	BBB Level	CYP2D6	Hepatotoxic	PPB
Daphnetin	0	4	3	FALSE	TRUE	FALSE
3-BP	1	5	4	FALSE	TRUE	FALSE
IA	3	5	4	FALSE	FALSE	FALSE

**Table 4:** The results of toxicity prediction.

Compound	Ames	Rat Oral LD_50_ (mg/kg_Body_Weight)	Rat Inhalational LC50 (mg/m^3^/h)	DTP Probability
Daphnetin	Non-mutagen	474.36	2305.35	0.61
3-BP	Non-mutagen	219.28	2599.56	0.53
IA	Non-mutagen	1209.33	2050.05	0.55

## Supplementary Materials

The following supporting information can be downloaded at: https://www.mdpi.com/article/10.3390/molecules27082447/s1, Figure S1: Schematic diagram of virtual screening procedure; Table S1: 2D diagram of compounds.

## Sample Availability

Samples of the compounds are available from the authors.
