# Supplementary material for: Discovery of a Novel Inhibitor Structure of Mycobacterium tuberculosis Isocitrate Lyase
Source: Molecules. 2022 Apr 11;27(8):2447. doi: 10.3390/molecules27082447 (PMC9026967; doi:10.3390/molecules27082447)
Supplement: Supplementary file 1 [file molecules-27-02447-s001.zip › molecules-1650529-supplementary.pdf]

Supplementary Informations

Discovery of a novel inhibitor structure of *Mycobacterium tuberculosis* isocitrate lyase

Changyuan Duan 1, Qihua Jiang 2, Xue Jiang1, Hongwei Zeng1, Qiaomin Wu1, Yang Yu1, and Xiaolan Yang 1,*

[1] Key Laboratory of Medical Laboratory Diagnostics of the Education Ministry, College of Laboratory Medicine, Chongqing Medical University, No.1, Yixueyuan Road, Yuzhong Dist, Chongqing 400016, China; 1033205960@qq.com(C.D.); 664282173@qq.com(X.J.); 1669178026@qq.com(H.Z.); 1140300146@qq.com(Q.W.); 2678741806@qq.com(Y.Y.).

[2] College of pharmacy, Chongqing Medical University, Chongqing 400016, China; 100877@cqmu.edu.cn(Q.J.).

* Correspondence: xiaolangyang666@cqmu.edu.cn(X.Y.); Tel.: (0086)-23-68485240.

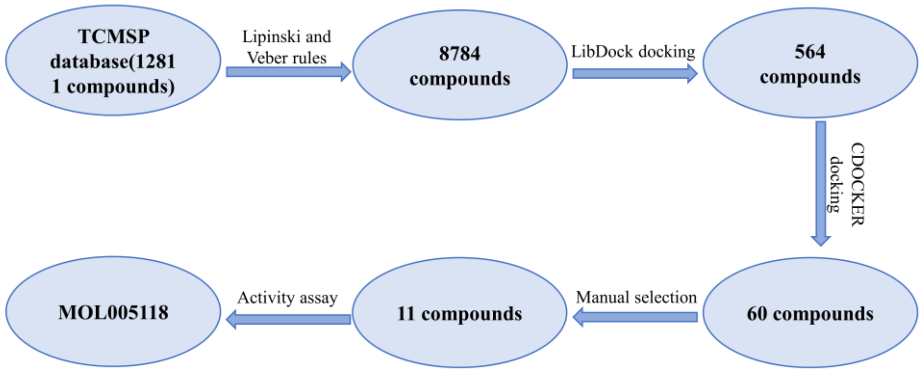

Figure S1. Schematic diagram of virtual screening procedure.

Table S1 2D diagram of compounds

Compound	2D diagram	Key residues
L-Ascorbic acid	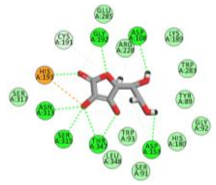	ASP108、GLY192、ASP153 HIS193、ASN313、SER315 THR347
4-Methylumbelliferone	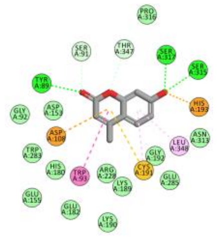	TYR89 TRP93 ASP108 CYS191 HIS193 SER317 SER315 LEU348

Quinic acid	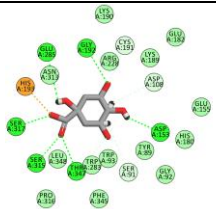	ASP153 GLY192 HIS193 GLU285 SER315 SER317 THR347
L-Shikimic acid	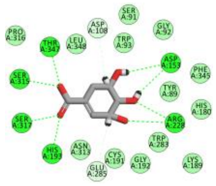	ASP153 HIS193 ARG228 SER315 SER317 THR347
Chelidonic acid	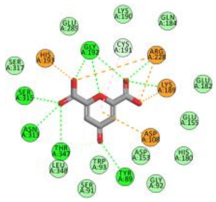	TYR89 ASP108 LYS189 CYS191 GLY192 HIS193 ARG228 ASN313 SER315 THR347
Gallic acid	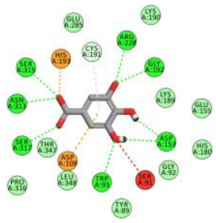	TRP93 ASP108 ASP153 GLY192 HIS193 ARG228 ASN313 SER315 SER317
Phosphatdic acid	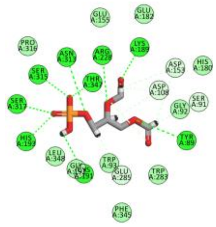	TYR89 LYS189 CYS191 HIS193 ARG228 ASN313 SER315 SER317 THR347
Methyl gallate	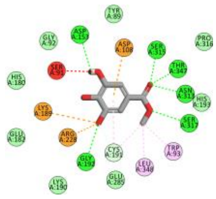	ASP108 ASP153 LYS189 GLY192 ARG228 ASN313 SER315 SER317 THR347
3-O-Ethy-L-ascorbic acid	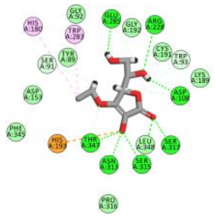	ASP108 HIS180 HIS193 ARG228 TRP283ASN313 SER315 SER317 THR347 LEU348
4-Deoxypyridoxine 5'-phosphate	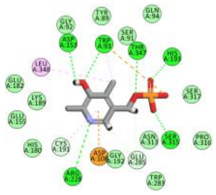	TRP93 ASP108 ASP153 HIS193 SER315 ARG228 THR347 LEU348

Daphnetin	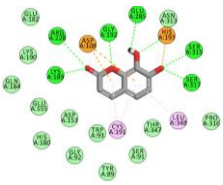	ASP108 LYS189 CYS191 GLY192 HIS193 ARG228 GLU285 SER315 SER317 LEU348
3-BP	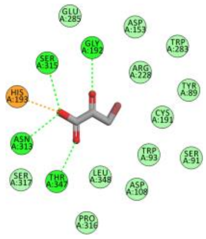	GLY192 HIS193 ASN313 SER315 THR347
IA	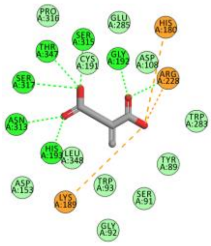	HIS180 LYS189 GLY192 HIS193 ARG228 ASN313 SER315 SER317 THR347
